# Where to Place Monitoring Sensors for Improving Complex Manufacturing Systems? Discussing a Real Case in the Food Industry

**DOI:** 10.3390/s23073768

**Published:** 2023-04-06

**Authors:** Miguel Rivas Pellicer, Mohamed Yoosha Tungekar, Silvia Carpitella

**Affiliations:** Department of Manufacturing Systems Engineering and Management, College of Engineering and Computer Science, California State University Northridge, 18111 Nordhoff Street, Northridge, CA 91330, USA

**Keywords:** sensor placement, Industry 4.0, manufacturing, MCDM, fuzzy DEMATEL, food industry

## Abstract

Industry 4.0 technologies offer manufacturing companies numerous tools to enhance their core processes, including monitoring and control. To optimize efficiency, it is crucial to effectively install monitoring sensors. This paper proposes a Multi-Criteria Decision-Making (MCDM) approach as a practical solution to the sensor placement problem in the food industry, having been applied to wine bottling line equipment at a real Italian winery. The approach helps decision-makers when discriminating within a set of alternatives based on multiple criteria. By evaluating the interconnections within the different equipment, the ideal locations of sensors are suggested, with the goal of improving the process’s performance. The results indicated that the system of electric pumps, corker, conveyor, and capper had the most influence on the other equipment which are then recommended for sensor control. Monitoring this equipment will result in the early discovery of failures, potentially also involving other dependant equipment, contributing to enhance the level of performance for the whole bottling line.

## 1. Introduction

The industrial revolution has driven companies to seek out cost-effective ways to improve manufacturing efficiency and product quality [1]. The fourth industrial revolution, Industry 4.0, has been fueled by rapid technological advances and is transforming people’s lives through the adoption of technologies such as AI, blockchain, AR, robotics, and IoT [2,3]. While Industry 4.0 has the potential to revolutionize traditional manufacturing processes, there are still challenges to overcome, such as economic and structural barriers [4,5]. Industry 4.0 can significantly impact business results, but preparation is necessary to effectively embrace the change [6].

Even though the impact of Industry 4.0 technologies on operations management has been widely recognized, a relevant gap still remains in the evolution of such fundamental manufacturing processes as monitoring and control, as observed by Guo et al. [7]. These processes may enormously benefit from addressing procedures capable of supporting informed decisions on where monitoring sensors should be placed throughout the production flow. Complex manufacturing plants are made of components and subsystems deeply interconnected with each other. It is hence reasonable to assume that sensors should be placed on those specific components characterized by higher degrees of interconnection with the other ones. This evidence has been sustained in previous research [8,9,10] with relation to the nodes of such complex systems as water distribution networks, and its validity can also be extended to the manufacturing sector. The use of various types of sensors available in Industry 4.0 allows for real-time monitoring and automated control systems, leading to sustainability improvements [11,12]. This represents the main motivation of this research in solving the problem of sensors placement with relation to complex manufacturing plants.

### 1.1. Sensors Placement in the Manufacturing Industry

The manufacturing industry has led the way regarding the implementation of cutting-edge sensor technology in their processes. In this type of industry, sensors are required to directly monitor changes in the products as the sophistication of material processing rises. Flexibility and automation are increasing demands in modern industries, and sensors are crucial decision variables that determine the process’s performance [13]. An optimized sensor placement is greatly influenced by several aspects, including cause-effect modeling, optimization benchmark, optimization approach, performance assessment and strategy implementation, all of which have a significant influence on the accuracy of monitoring the production system. Numerous studies have been carried out and have yielded several encouraging findings. As highlighted by He et al. [14], key information regarding the best sensor placement for diagnosing a production system has been addressed in the literature.

The basis for diagnosing manufacturing systems is modeling the cause-and-effect relationship between system faults and sensor readings [15]. Various optimization strategies proposed in the existing literature make use of gradient-based search algorithms to find the best sensor distribution in multi-fixture assembly systems [16,17,18]. With the same objective, other authors employed exchange algorithms to diagnose the sources of dimensional variation in assembly processes [19,20,21]. Furthermore, intelligent optimization algorithms were developed: Li and Jin [22] created an integrated method by fusing a pre-processing algorithm and a greedy algorithm to optimize sensor placement and detect anomalies during a hot forming process; Lu et al. [23] presented the ideal position for acoustic emission using an updated Particle Swarm Optimization (PSO) technique; and numerous ideal sensor placements have been created to optimize several competing assessment criteria using the principles of Genetic Algorithms (GAs) [24,25,26,27].

Early studies on the optimization of sensor placement exclusively included single objective optimization and sensor homogeneity [28]. The fault transmissibility is considered for the multi-station manufacturing system, but the sensor property is left out. Most of the recent research aims to propose approaches that take into account the many objectives involved in sensor arrangement as well as the heterogeneity of sensor properties. Different implementing plans are required for sensor placement and have their own unique characteristics for each different application scenario [14].

### 1.2. Multi-Criteria Methods in Industry 4.0

Multi-Criteria Decision-Making (MCDM) methods are commonly used in the engineering practice as they are effective and flexible tools capable of aiding in decision-making when discriminating within a set of alternatives under multiple criteria is required [29,30]. MCDM methods have the ability to use both quantitative and qualitative data, which can be translated into numerical values using specific linguistic scales [31]. This flexibility allows these methods to be applied to various fields, including energy [32,33], production quality [34,35], environment [36,37,38], healthcare [39,40], and others.

Among the several MCDM techniques proposed in the literature, the DEcision-MAking Trial and Evaluation Laboratory (DEMATEL) [41] is a particularly useful tool in our specific context of research, given its capability of developing a structural framework displaying the causal relationships among decision-making elements interdependent with each others [42]. DEMATEL has been successfully used to address Industry 4.0 applications. For example, Asadi et al. [42] used DEMATEL to assess the significance of factors related to IoT adoption in IT procurement for manufacturing companies in Malaysia. Torbacki and Kijewska [43] proposed a DEMATEL application to determine the most significant factors improving sustainability for logistics and manufacturing processes. DEMATEL has also been applied to predictive maintenance management for complex service systems monitored by sensors [44,45] and to evaluate blockchain adoption enablers in sustainable supply chain management [46]. DEMATEL has been integrated with other methodologies, such as Interpretive Structural Modeling (ISM) [47], to analyze barriers for the implementation of a circular economy in the automotive industry [48] and with the Best Worst Method (BWM) [49] to assess human resource management challenges related to Industry 4.0 in the Indian automotive sector [50]. As previously discussed, MCDM methods can effectively rely on qualitative evaluations as input data and DEMATEL makes no exception. This is the reason why extending the DEMATEL framework in a fuzzy environment is particularly suitable in the context of the present research, since we are going to deal with expert evaluations. The efficacy of this has been widely demonstrated in the literature, where a diverse plethora of fuzzy DEMATEL solutions have been proposed in the context of Industry 4.0 [51,52,53].

### 1.3. Focus on the Food Industry

A food system, as defined by the Food and Agriculture Organization, is a group of individuals and the actions they engage in order to produce, gather, process, distribute, consume, and dispose of food items [54]. The complexity of food systems, which include many processes, value chains, players, and interactions, is implied by this. When integrated into the architecture of the food supply chain, sensors are one of the data acquisition technologies that make it possible to collect, process, store, and analyze data [54]. The use of sensors in the food industry is in its early stages. In recent times, wireless sensors have been utilized for monitoring and controlling the quality of food products during processing. Although they cater to short-term business requirements, the financial advantages of wireless sensing and the broader “machine-to-machine” approach are noteworthy [55]. In this context, an important issue refers to the compatibility of sensors with different types of food and drink. Watson et al. [56] noted that the type of sensor used can depend on a variety of factors, such as the physical and chemical properties of the food, the desired level of accuracy, and the environmental conditions of the manufacturing process. For instance, some sensors may not be suitable for use with acidic or corrosive foods, while others may be affected by high temperatures or humidity. Therefore, careful consideration and testing is required to ensure that the sensors used are compatible with the specific food or drink being manufactured, as also pointed out by Wang et al. [57] with relation to agricultural products. The authors emphasize the need for continued research and development in the field of wireless sensors to improve the compatibility and accuracy of sensors for use in the agriculture and food industry. The food business is now undergoing significant changes in response to customer demands, which require an ever-growing range of food items with high quality standards in addition to worries about health and safety. Better quality assurance techniques are required by all parties to meet consumer expectations and establish a competitive edge [58]. It is crucial to be able to obtain precise and thorough information about the environment, and combine the depth data from several sensors. This demands the proper positioning of sensors for better accuracy.

### 1.4. Contribution of Research

The main objective of research is to propose an MCDM approach aimed at evaluating interconnections within the network of components and subsystems belonging to manufacturing facilities focusing on the food industry.

According to the best knowledge of the authors, such a research area has not yet been satisfactorily developed at the current state of the art, with special regard to the food industry. While various works have been focusing on the use of sensors to monitor the quality of finished products [59] and to detect the presence of potential contaminant agents in food [60], our approach aims to study the interconnections existing within the network of equipment deputed to food production in order to determine the most favorable positions of sensors. Monitoring a restricted set of components with priority (i.e., those components that are more interdependent with other ones) also increases the probability of detecting failures occurring—or about to occur—in other parts of the manufacturing process. This approach aims to improve the state of the same equipment through prompt maintenance while simultaneously limiting the number of sensors to be installed along with the related cost. The choice of the food sector lies on its inarguable importance in manufacturing.

However, the proposed approach may be extended also to other sectors of activities. This flexibility can be achieved since we propose an evaluation based on a MCDM approach integrating expert opinions into a mathematical model.

## 2. Material and Methods

### 2.1. Choice of a Suitable MCDM Method

We have previously noted that DEMATEL has the ability to develop a structural framework displaying the causal relationships within a decision-making set of elements that are interdependent with one another. This is a result that other MCDM methods are not capable of achieving. Furthermore, as discussed in Section 1.2, the fuzzy evolution of the DEMATEL technique can effectively deal with linguistic evaluations provided by a panel of decision-makers. These evaluations can be indeed represented by fuzzy numbers instead of crisp values, something that guarantees to achieve more precise results [61]. For these reasons, the fuzzy DEMATEL is herein proposed to support the problem of sensors placement in in the food industry, specifically in an Italian winery, given its proven capability of:

- involving a panel of decision-makers with proven experience in the field and integrating elements of the fuzzy set theory to effectively treat human evaluations of preference and produce meaningful results;
- analyzing dependence relations existing within a set of decision-making elements, in our case components and subsystems of manufacturing plants as candidates for locating monitoring sensors;
- establishing that restricted set of elements characterized by the higher degree of interdependence, then likely impacting all the other elements (in other words, this means that locating a sensor in proximity of a component with an associated higher degree of interdependence increases the probability of detecting failures potentially involving other related components).

We specify that proposing methodological innovations is not a goal of this research, since the traditional framework of the fuzzy DEMATEL is perfectly suitable to deal with the problem of interest. However, the application of MCDM to support the problem of sensors placement with relation to core manufacturing plants seems to be quite an unexplored area in the existing literature. Given that MCDM methods are extremely valuable tools to support management in dealing with core decision-making problems, we aim to bridge this gap in the area of monitoring and control. Furthermore, to the best of the authors’ knowledge, this is the first time that the fuzzy DEMATEL is proposed to solve the problem object of research and also in the specific context of the food industry.

A description of the fuzzy DEMATEL technique is provided in the following Section 2.2, while Section 2.3 provides context details about a real company operating in the sector under analysis.

### 2.2. Fuzzy DEMATEL: Methodological Details

It is first necessary to carefully describe the context of analysis as well as the goal of the decision-making problem, i.e., in our case the prioritization of equipment for suitable locations for sensors. Such equipment will constitute the decision-making element of the problem. It is also preliminarily necessary to involve an expert (or a panel of decision-makers) capable of formulating judgments related to the existence of dependence between pairs of elements along with the intensity of such a dependence, to be established according to a linguistic scale. Linguistic evaluations will be treated as fuzzy numbers, as explained next. The fuzzy DEMATEL procedure can be then applied as a sequence of the following methodological steps.

1. Design evaluation procedures and a corresponding fuzzy linguistic scale. In order to handle the ambiguity of human thought, this phase requires replacing conventional measurement scales with a fuzzy linguistic scale. In order to estimate the degree of interacting influence between variables, Triangular Fuzzy Numbers (TFNs), e.g., ñ=(l,m,u) are typically used: (0,0,0.25) triplet denotes that no influence exists between two elements (NO), (0,0.25,0.5) triplet denotes a very low (VL) influence between two elements, (0.25,0.5,0.75) triplet denotes a low (L) influence between two elements, (0.5,0.75,1) triplet denotes a high (H) influence between two elements, and (0.75,1,1) triplet denotes a very high (VH) influence between two elements.
2. Elicit evaluations from each expert to generate an input fuzzy direct-relation matrix for each expert. The set of decision-making elements to be pairwise compared is denoted as $C=C_{i}|i=1,2,\ldots ,n$. These comparisons are expressed in the form of linguistic judgments by a panel composed of *p* experts or even a single decision-maker (in this last case, p=1). The expressed linguistic judgments reflect the degree of influence between pairs of elements to be attributed by using the fuzzy linguistic scale previously formalized. Furthermore, these judgments will be collected in fuzzy matrices. Being *p* the number of interviewed experts, *p* fuzzy matrices are obtained ($\tilde{Z}{}^{\left(1\right)},\;$$\tilde{Z}{}^{\left(2\right)},\ldots ,\;$$\tilde{Z}{}^{\left(p\right)}$). The generic fuzzy matrix $\tilde{Z}{}^{\left(k\right)}$ represents the input fuzzy direct-relation matrix determined by the k^*th*^ expert when pairwise comparing elements $C_{i}$. We can notice as TFNs translated from linguistic evaluations are in the cells of the matrix, that can be expressed as follows:
  (1) $${\tilde{Z}}^{\left(k\right)}=\left[\begin{array}{cccc} \left(0,0,0\right) & {\tilde{z}}_{12}^{\left(k\right)} & \ldots & {\tilde{z}}_{1n}^{\left(k\right)}\\ {\tilde{z}}_{21}^{\left(k\right)} & \left(0,0,0\right) & \ldots & {\tilde{z}}_{2n}^{\left(k\right)}\\ \vdots & \vdots & \ddots & \vdots \\ {\tilde{z}}_{n1}^{\left(k\right)} & {\tilde{z}}_{n2}^{\left(k\right)} & \ldots & \left(0,0,0\right) \end{array}\right],k=1,2,\ldots ,p;$$
  being $\tilde{z}{}_{ij}^{\left(k\right)}=\left(l_{ij}^{\left(k\right)},m_{ij}^{\left(k\right)},u_{ij}^{\left(k\right)}\right)$, and setting the TFN (0,0,0) for (i=1,2,…,n).
3. Aggregate the previously elicited matrices into a single fuzzy direct-relation matrix $\tilde{Z}$. The input fuzzy direct-relation matrices defined by each expert during the previous step can be aggregated using various aggregation methods, e.g., the arithmetic mean. The result will be a single matrix $\tilde{Z}$ that synthesizes the judgments provided by the involved decision-making group.
4. Obtain the normalized fuzzy direct-relation matrix $\tilde{X}$. The normalized fuzzy direct-relation matrix $\tilde{X}$ can be indicated as:
  (2) $$\tilde{X}=\left[\begin{array}{cccc} {\tilde{x}}_{11} & {\tilde{x}}_{12} & \ldots & {\tilde{x}}_{1n}\\ {\tilde{x}}_{21} & {\tilde{x}}_{22} & \ldots & {\tilde{x}}_{2n}\\ \vdots & \vdots & \ddots & \vdots \\ {\tilde{x}}_{n1} & {\tilde{x}}_{n2} & \ldots & {\tilde{x}}_{nn} \end{array}\right].$$
  Elements of $\tilde{X}$ are obtained by normalizing the elements of matrix $\tilde{Z}$ as follows:
  (3) $${\tilde{x}}_{ij}=\frac{{\tilde{z}}_{ij}}{r}=\left(\frac{l_{ij}}{r},\frac{m_{ij}}{r},\frac{u_{ij}}{r}\right),$$
  where
  (4) $$r=\max_{ij}\left\{\max_{i}\sum_{j=1}^{n}u_{ij},\max_{j}\sum_{i=1}^{n}u_{ij}\right\},1\leq i,j\leq n.$$
5. Achieve the fuzzy total-relation matrix $\tilde{T}$. In traditional DEMATEL (crisp version of the method), the total-relation matrix *T* associated to a given normalized direct-relation matrix X is obtained as:
  (5) $$T=\lim_{w\to \infty }(X+X^{2}+\ldots +X^{w})=X{\left(I-X\right)}^{-1},$$
  being *I* the identity matrix. The convergence of this series is guaranteed by the performed normalization. In fuzzy DEMATEL, this operation is made element-wise with respect to the components of the TFNs in matrix $\tilde{X}$ by using the fuzzy operator ⊕, as follows:
  (6) $$\tilde{T}=\lim_{w\to \infty }\left(\tilde{X}⊕{\tilde{X}}^{2}⊕\ldots ⊕{\tilde{X}}^{w}\right).$$
  The convergence of the three series involved is guaranteed by the normalization Formula (3).
6. Obtain the crisp total-relation matrix. Fuzzy linguistic values have to be finally defuzzified into crisp values. With this aim, it is possible to use one of the defuzzification methods available in the literature, for example the Converting Fuzzy Data into Crisp Scores (CFSC) algorithm, proposed by Opricovic and Tzeng [62] to obtain a crisp value of total-relation matrix (i.e., matrix *T*).
7. Set the threshold value. Calculating the internal relations matrix requires a previously established suitable threshold value. The Network Relationship Map (NRM) is plotted by neglecting partial relations. Only relationships whose values in matrix T exceed the threshold value are shown in the NRM. It is sufficient to determine the average values of the matrix T in order to determine the threshold value for relations. Once the threshold intensity has been established, all values in matrix T that are below the threshold value are set to zero, meaning that the causal relationship indicated above is not taken into account.
8. Produce a final cause-and-effect diagram. To identify the causal connections and interactive influences between the various elements, results derived from the procedure are eventually depicted in a diagram showing cause-and-effect relationships. The sum of each row and each column of *T* has to be computed. The sum of rows (*D*) and columns (*R*) can be calculated as follows:
  (7) $$D=\sum_{j=1}^{n}T_{ij};$$
  (8) $$R=\sum_{i=1}^{n}T_{ij}.$$
  The values of (D+R) and (D−R) show, respectively, the system’s overall importance of element *i* and its net impacts as a result of component *i*. By aligning the values of (D+R) on the horizontal axis and (D−R) on the vertical axis, the cause-and-effect diagram is created. The coordinate system determines the position of each factor and how it interacts with a point in the space (D+R,D−R).

### 2.3. The Bottling Line of an Italian Winery

The choice of the food industry as a main sector of application aims to cover two main aspects. First, the problem of sensor placement does not appear to be satisfactorily explored in the literature for these types of manufacturing systems. As already observed, food and beverage manufacturing is one of the largest manufacturing sectors worldwide. As affirmed by Owusu-Apenten and Vieira [63], the amount of revenue generated and jobs created demonstrate the economic importance of the food industry for various nations. This is the main reason why the relevance of this sector to our research problem is unarguable. The second aspect refers to the possibility of dealing with production lines for which the relations of dependence manipulated by the fuzzy DEMATEL are going to be crucial. This aspect also invites the flexibility of our approach, which can be easily extended to other industrial contexts.

The company selected for our case study is an Italian winery which produces, bottles and commercializes different types of wines and sparkling wines. The industrial settlement consists of buildings, warehouses, and delimited external areas used for vehicle circulation and the placement of silos and plants. External squares in the industrial settlement host warehouses built for the storage of agricultural materials and vehicles. A preliminary contextual analysis has been led through several brainstorming sessions with the responsible of the safety and security system of the company. Business processes have been analyzed in detail in order to establish the boundary of our analysis. They can be synthesized through the following main work stages: delivering grape; weighing and sampling; receiving white/black grapes; mashing; wine-making; storing and transferring; ripening; stabilization; aging; bottling; stocking; marketing finished products. We selected the bottling as one of the core processes taking place in the winery, where a complex production line made of interconnected plants is continuously operating. The line dedicated to bottling is located next to the stabilization department. The still wines to be bottled are stabilized in stainless steel tanks before being transferred to the aforementioned bottling line after filtration and micro-filtration. The winery also processes sparkling wines using special autoclaves. We specify that the area including stabilization and bottling contains work equipment, tanks, bottling line, autoclaves for sparkling wine, micro-filtration systems and necessary technical services, as well as a conservation and storage space for bottled wines.

Table 1 classifies the different jobs involved in the bottling process, along with their description, the related equipment and materials. Each job has been identified with a lowercase letter, while the equipment needed to perform that job has been identified with the same (capital) letter and numbered accordingly. For jobs performed by single equipment, that equipment has not been numbered (e.g., equipment *A*, *E*, *F*, *G*, *J*, *K*, *L*). A total of 19 pieces of equipment have been identified and formalized in the fourth column of Table 1. We exclude from our analysis equipment *K* and *L*, as they include complementary machines and/or components used for maintaining, cleaning and sanitizing the bottling plant, not being eligible for sensor placement. In the next section, we will apply the fuzzy DEMATEL to the set of the remaining 17 pieces of equipment ($C=C_{i}|i=1,2,\ldots ,17$).

## 3. Results

The data required for the implementation of the practical application was gathered throughout multiple brainstorming sessions conducted with two experts (p=2), specifically, the technical consultant and the safety and security system administrator of the company. Linguistic evaluations provided by experts have been translated to TFN according to the scale previously defined and recalled in Table 2. There was a significant level of consensus between the two stakeholders, leading to a unanimous agreement on the linguistic evaluations presented in Table 3. To explain this process in greater detail, involving multiple experts broadly allows us to gather diverse perspectives on the data, which can lead to more accurate results. Each expert compiles a distinct matrix, as described in step two of the fuzzy DEMATEL methodology. These matrices must then be combined, as outlined in step three, to create a single input matrix. This aggregation process generally affects the TFN values within the input matrix. In our case study, despite involving two experts, they arrived at identical evaluations, resulting in a single input matrix (Table 3). This is why, even though two different experts were consulted, we only present a single input matrix, as there was no difference in the evaluations of the two experts.

The input fuzzy direct-relation matrix has been normalized by means of the operation of linear conversion described in step three of the fuzzy DEMATEL procedure. The fuzzy total-relation matrix has been consequently achieved by applying the fourth step of the procedure. The process of obtaining the fuzzy total-relation matrix involves performing the following calculations in order: (1) calculate the inverse of the normalized input fuzzy direct-relation matrix, (2) subtract this inverse from the identity matrix, and (3) multiply the resulting matrix with the normalized input fuzzy direct-relation matrix. We specify that the input fuzzy direct-relation matrix, the normalized input fuzzy direct-relation matrix and the fuzzy total-relation matrix are not herein reported for the sake of space, while the crisp total-relation matrix T, obtained via the CFCS method [62], is reported in Table 4.

To determine the internal relations matrix, it is necessary to calculate a threshold value. This threshold value is used to determine which relations should be included in the NRM. Relations with values that are lower than the threshold in the crisp total-relation matrix are disregarded and not plotted in the NRM. To determine the threshold value for relations, the average values of matrix *T* are calculated. In this study, the threshold value was found to be 0.109, that is the average of the numerical values of the matrix reported in Table 4. As a result, all values in matrix *T* that are below 0.109 were set to zero, meaning that their causal relation is not taken into account. This model of significant relations is presented in Table 5. The threshold value serves as a criterion for identifying which relationships are deemed important and relevant enough to be included in the NRM.

The next step consists of calculating the sum of rows (*D*) and columns (*R*) of the crisp total-relation matrix (Table 4). Subsequently, the values of (D+R) and (D−R) can be determined based on the values of *D* and *R*. As already explained, (D+R) represents the degree of significance of equipment *i* in the overall system, while (D−R) indicates the net impact that equipment *i* has on the system. These values provide a clear picture of the importance and effect of equipment *i* on the system.

**Figure 1:**
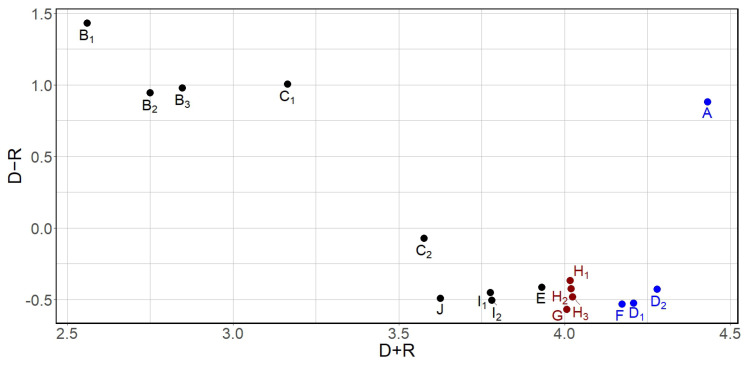
Final cause-and-effect diagram.

The conclusive results are presented in Table 6, and the model of significant relations is illustrated in Figure 1. This model is depicted as a diagram where the values of (D+R) are displayed on the horizontal axis and the values of (D−R) on the vertical axis. The position and interaction of each factor in the (D+R,D−R) coordinate system is determined by the values of (D+R) and (D−R).

## 4. Discussion

The diagram reported in Figure 1 provides an evaluation of each piece of equipment based on the following characteristics. The horizontal vector (D+R) represents the significance of each equipment in the overall system. Not only does this value indicate the impact of equipment *i* on the system, but also takes into account the impact of all the other equipment on equipment *i*. In terms of importance, equipment *A* holds the highest rank, followed by equipment $D_{2}$, equipment $D_{1}$, and equipment *F* in descending order. The vertical vector (D−R) represents the extent to which a single equipment impacts the system. Generally, a positive value of (D−R) indicates a causal variable and a negative value represents an effect. In this study, equipment *A*, $B_{1}$, $B_{2}$, $B_{3}$, $C_{1}$ are identified as causal variables, whereas equipment $C_{2}$, $D_{1}$, $D_{2}$, *E*, *F*, *G*, $H_{1}$, $H_{2}$, $H_{3}$, $I_{1}$, $I_{2}$, *J* are considered effects.

A practical solution to the problem of sensor placement is to suggest ideal positions for sensors based on the results of the fuzzy DEMATEL approach. The analysis showed that equipment *A*, $D_{2}$, $D_{1}$, and *F* (respectively, system of electric pumps, corker, conveyor, and capper, highlighted in blue in Figure 1) have a higher degree of influence on all the other equipment in the wine bottling line, making them favorable candidates for sensor control. Depending on the available budget, the company may refer to Figure 1 to take into account the possibility to install sensors monitoring other equipment that, in order, present higher values of D+R (i.e., prominence), for example equipment $H_{1}$, $H_{2}$, $H_{3}$, and/or *G* (respectively, shaper, canner, gluer, and labeller, highlighted in red in Figure 1). As said, the number of sensors installed will depend on the company’s budget, and controlling the most interdependent equipment will lead to significant improvements in the overall process. Monitoring more equipment will result in better maintenance optimization and performance improvements. This structured procedure will enable the company to make an informed decision to achieve a good trade-off between improving monitoring and control of equipment and minimizing sensor installation costs. Apart from maintenance optimization and performance improvements, careful monitoring leads to increased efficiency and productivity, as noted by various authors in the literature [64,65]. By identifying potential failures or issues early on, companies can proactively address them before they escalate into major problems that could result in downtime, loss of revenue, and even safety hazards. Some important practical considerations for the bottling process include the critical importance of the job of starting up the product transfer plant, followed by the jobs of corking, canning, and labeling, in that order. The equipment used for these jobs has a direct impact on the functioning of all the other equipment used for performing the remaining jobs. In other terms, any failures or malfunctions in these machines could lead to delays or defects in the finished product, causing delays and even loss of revenue. This is the reason why the early diagnosis of failures affecting these equipment is important to prevent failures potentially involving other machines. Therefore, it is essential to continuously monitor and diagnose the performance of these machines to ensure that they are operating at their optimal level. The use of fuzzy DEMATEL can aid in identifying the interrelationships between different pieces of equipment and their impact on the overall process. By determining which piece of equipment has a positive value of D−R relation, companies can focus on monitoring and diagnosing these machines to prevent failures from affecting other operations. The fuzzy DEMATEL application results also show that the equipment used for the starting-up product transfer plant has a positive value of D−R (relation), indicating that it directly affects the other operations [66]. The remaining jobs are mostly influenced by other jobs, as the related equipment has a negative value of relation. By identifying equipment with a negative value of relation, companies can take measures to improve the performance and reliability of these machines, potentially reducing their impact on other operations.

The benefits of monitoring equipment can be significant. However, the costs and potential benefits of sensor installation must be carefully weighed, as monitoring non-critical equipment may not provide significant benefits and may result in unnecessary costs [67]. Companies must achieve a good trade-off balancing these two aspects. By using the structured procedure proposed in the present research, companies can make informed decisions about which equipment to monitor and the most cost-effective methods for doing so. This approach can help companies achieve a good trade-off between improving monitoring and control of equipment and minimizing sensor installation costs. The proposed method is highly adaptable and can be applied to any other industrial process and to companies operating in different sectors. The results of this approach can be used as an organizational best practice and will be incorporated into the company’s documentation for future reference.

While fuzzy DEMATEL has been proven to be a useful tool for decision-making in complex systems, it also has limitations that must be considered when applying it to real-world problems, similarly to sensor placement in the food industry. One limitation is that fuzzy DEMATEL requires a large amount of input data, and the accuracy of the results is highly dependent on the quality of the data. In addition, the complexity of the system being analyzed can make it difficult to identify all the relevant variables and relationships. Another limitation is that fuzzy DEMATEL assumes that the relationships between variables are linear, which may not always be the case in real-world systems. These limitations highlight the need for careful validation when applying fuzzy DEMATEL to real-world problems.

## 5. Conclusions

In this research, the main objective is to propose an MCDM approach aimed at evaluating interconnections within the network of components and subsystems belonging to manufacturing facilities focusing on the food industry. To such an aim, a MCDM approach integrating fuzzy information has been applied to the production line of a company operating in the food industry. Specifically, the fuzzy DEMATEL method has been proposed to model a problem of sensor placement by dealing with linguistic evaluations of input and exploring the significance and the impact of various equipment. This was with regards to the consideration of machines for sensor-friendly locations belonging to the wine bottling line of a real wine-making company located in Italy.

The methodology evaluation reveals that the system of electric pumps, corker, conveyor, and capper in the bottling line have a significant influence on the functioning of other equipment. Thus, installing sensors on these machines could be highly beneficial for improving overall process efficiency. However, the decision of which equipment to install sensors on should also consider the company’s budget limitations. In addition to these influential machines, other equipment with lower prominence values can also be considered for sensor placement. The suggested order of installation should be based on the prominence values associated with each equipment, with the most influential machines being given priority. By carefully selecting and placing sensors on the most interdependent pieces of equipment, the bottling line can significantly improve its performance and maintenance optimization. This research directs a data-driven approach to make informed decisions for companies willing to enhance equipment monitoring and control. It also highlights the use of the fuzzy DEMATEL method in an area where the scope of other MCDM approaches is limited, such as the problem of sensor placement in the food industry.

Future research could explore the possibility of applying MCDM in other areas or aspects of the food industry. For example, it could be used to improve the management of perishable food products by evaluating multiple relevant factors such as temperature, humidity, quality, light, and safety. By using MCDM, it may be possible to enhance food supply chain efficiency, reduce food waste, and improve food safety.

## Figures and Tables

**Table 1:** Bottling process: jobs, descriptions, equipment, materials.

ID	Jobs	Descriptions	Equipment	Materials
a.	Starting-up product transfer plant	Starting-up compressor; starting-up nitrogen machine; starting-up heating decant system.	$A_{}$ System of electric pumps	Wine
b.	Preparing empty bottles	Arranging empty bottles (depalletizer); washing empty bottles with micro-filtered water.	$B_{1}$ Depalletizer; $B_{2}$ Rinser; $B_{3}$ Osmosis system	Empty bottles
c.	Wine filling	Preparing input; checking cartridge; micro-filtrating wine.	$C_{1}$ Filtering system; $C_{2}$ Filler	Cartridge
d.	Corking	Loading corks; corking.	$D_{1}$ Conveyor; $D_{2}$ Corker	Corks
e.	Washing/drying	Introducing water and air.	$E_{}$ Drywasher	-
f.	Encapsulating	Loading capsule; encapsulation.	$F_{}$ Capper	Capsule
g.	Labelling	Loading labels; labelling.	$G_{}$ Labeller	Labels
h.	Canning	Loading cartons; forming boxes; canning; pasting.	$H_{1}$ Shaper; $H_{2}$ Canner; $H_{3}$ Gluer	Cartons
i.	Palletizing	Forming pallet; wrapping pallet.	$I_{1}$ Palletizer; $I_{2}$ Winder	Platforms, Films
j.	Handling pallets	Loading/unloading pallets.	$J_{}$ Moving system	-
k.	Maintaining and controlling	Interventions to be led in accordance with the use and maintenance manual.	$K_{}$ Complementary work equipment for maintenance	Spare parts
l.	Cleaning and disinfecting	Cleaning working areas and machines.	$L_{}$ Complementary work equipment for sanitization	Detergents, Water

**Table 2:** Adopted evaluation scale translating linguistic evaluations to TFN.

ID	Linguistic Evaluation	Semantic Meaning	Corresponding TFN
1	NO	No influence	0, 0, 0.25
2	VL	Very low influence	0, 0.25, 0.5
3	L	Low influence	0.25, 0.5, 0.75
4	H	High influence	0.5, 0.75, 1
5	VH	Very high influence	0.75, 1, 1

**Table 3:** Linguistic evaluations of influence unanimously attributed by the interviewed experts.

	$A_{}$	$B_{1}$	$B_{2}$	$B_{3}$	$C_{1}$	$C_{2}$	$D_{1}$	$D_{2}$	$E_{}$	$F_{}$	$G_{}$	$H_{1}$	$H_{2}$	$H_{3}$	$I_{1}$	$I_{2}$	$J_{}$
$A_{}$	NO	VH	VH	VH	VH	VH	VH	VH	VH	VH	VH	VH	VH	VH	VH	VH	NO
$B_{1}$	L	NO	VH	VH	VH	L	L	L	L	L	L	L	L	L	L	L	VH
$B_{2}$	L	NO	NO	VH	VH	VH	L	L	L	L	L	L	L	L	L	L	L
$B_{3}$	H	NO	VH	NO	VH	H	H	L	L	L	L	L	L	L	L	L	L
$C_{1}$	H	NO	VH	VH	NO	VH	VH	H	H	H	L	L	L	L	L	L	L
$C_{2}$	L	NO	VH	L	H	NO	H	H	H	H	H	VL	VL	VL	VL	VL	L
$D_{1}$	H	NO	NO	L	L	H	NO	VH	VH	VH	VH	L	L	L	VL	VL	L
$D_{2}$	H	NO	NO	NO	L	H	H	NO	VH	VH	VH	H	H	H	L	L	L
$E_{}$	L	NO	NO	NO	NO	H	H	VH	NO	VH	H	H	H	H	L	L	L
$F_{}$	L	NO	NO	NO	NO	L	H	VH	VH	NO	VH	H	H	H	H	H	L
$G_{}$	L	NO	NO	NO	NO	L	H	VH	H	VH	NO	H	H	H	L	L	L
$H_{1}$	L	NO	NO	NO	NO	VL	H	H	L	H	H	NO	VH	VH	VH	VH	VH
$H_{2}$	L	NO	NO	NO	NO	VL	H	H	L	H	H	H	NO	VH	VH	VH	VH
$H_{3}$	L	NO	NO	NO	NO	VL	H	H	L	H	H	H	H	NO	VH	VH	VH
$I_{1}$	L	NO	NO	NO	NO	L	H	L	L	L	L	H	H	H	NO	VH	VH
$I_{2}$	L	NO	NO	NO	NO	L	H	L	L	L	L	H	H	H	H	NO	VH
$J_{}$	NO	H	NO	NO	NO	NO	L	L	L	L	L	H	H	H	VH	VH	NO

**Table 4:** Crisp total-relation matrix (*T*) obtained via the CFCS method [62].

	$A_{}$	$B_{1}$	$B_{2}$	$B_{3}$	$C_{1}$	$C_{2}$	$D_{1}$	$D_{2}$	$E_{}$	$F_{}$	$G_{}$	$H_{1}$	$H_{2}$	$H_{3}$	$I_{1}$	$I_{2}$	$J_{}$
$A_{}$	0.105	0.089	0.111	0.112	0.120	0.162	0.191	0.190	0.180	0.190	0.186	0.180	0.182	0.184	0.175	0.176	0.123
$B_{1}$	0.110	0.026	0.102	0.103	0.109	0.113	0.135	0.133	0.126	0.133	0.130	0.127	0.128	0.129	0.123	0.124	0.145
$B_{2}$	0.105	0.027	0.040	0.099	0.105	0.134	0.129	0.127	0.120	0.127	0.124	0.120	0.121	0.122	0.116	0.117	0.113
$B_{3}$	0.121	0.028	0.099	0.043	0.107	0.125	0.146	0.131	0.124	0.131	0.128	0.124	0.125	0.126	0.119	0.120	0.115
$C_{1}$	0.127	0.030	0.102	0.103	0.053	0.143	0.165	0.153	0.146	0.153	0.137	0.131	0.132	0.133	0.126	0.127	0.122
$C_{2}$	0.102	0.026	0.095	0.071	0.091	0.074	0.138	0.138	0.132	0.138	0.135	0.102	0.103	0.104	0.097	0.098	0.108
$D_{1}$	0.119	0.027	0.039	0.068	0.075	0.121	0.097	0.155	0.148	0.155	0.152	0.122	0.123	0.124	0.103	0.103	0.112
$D_{2}$	0.121	0.028	0.038	0.040	0.074	0.122	0.147	0.103	0.151	0.159	0.156	0.140	0.142	0.143	0.122	0.123	0.117
$E_{}$	0.101	0.026	0.033	0.034	0.041	0.115	0.139	0.151	0.086	0.151	0.138	0.133	0.134	0.135	0.115	0.116	0.112
$F_{}$	0.103	0.026	0.032	0.034	0.041	0.103	0.142	0.154	0.146	0.097	0.151	0.136	0.138	0.139	0.132	0.133	0.115
$G_{}$	0.100	0.025	0.031	0.033	0.039	0.099	0.137	0.149	0.130	0.149	0.090	0.132	0.133	0.134	0.113	0.114	0.110
$H_{1}$	0.102	0.028	0.031	0.033	0.039	0.087	0.141	0.141	0.119	0.141	0.139	0.091	0.150	0.151	0.145	0.147	0.142
$H_{2}$	0.101	0.027	0.031	0.033	0.039	0.086	0.140	0.140	0.118	0.140	0.138	0.136	0.091	0.150	0.144	0.145	0.140
$H_{3}$	0.100	0.027	0.031	0.033	0.039	0.085	0.139	0.138	0.117	0.138	0.137	0.135	0.136	0.091	0.143	0.144	0.139
$I_{1}$	0.096	0.026	0.030	0.032	0.037	0.096	0.134	0.119	0.112	0.119	0.117	0.129	0.130	0.131	0.080	0.139	0.135
$I_{2}$	0.096	0.026	0.030	0.032	0.037	0.095	0.132	0.118	0.111	0.118	0.116	0.128	0.129	0.130	0.125	0.080	0.133
$J_{}$	0.065	0.068	0.028	0.030	0.035	0.063	0.114	0.113	0.106	0.113	0.111	0.125	0.126	0.127	0.134	0.135	0.077

**Table 5:** Crisp total-relation matrix considering the threshold value.

	$A_{}$	$B_{1}$	$B_{2}$	$B_{3}$	$C_{1}$	$C_{2}$	$D_{1}$	$D_{2}$	$E_{}$	$F_{}$	$G_{}$	$H_{1}$	$H_{2}$	$H_{3}$	$I_{1}$	$I_{2}$	$J_{}$
$A_{}$	0.000	0.000	0.111	0.112	0.120	0.162	0.191	0.190	0.180	0.190	0.186	0.180	0.182	0.184	0.175	0.176	0.123
$B_{1}$	0.110	0.000	0.000	0.000	0.000	0.113	0.135	0.133	0.126	0.133	0.130	0.127	0.128	0.129	0.123	0.124	0.145
$B_{2}$	0.000	0.000	0.000	0.000	0.000	0.134	0.129	0.127	0.120	0.127	0.124	0.120	0.121	0.122	0.116	0.117	0.113
$B_{3}$	0.121	0.000	0.000	0.000	0.000	0.125	0.146	0.131	0.124	0.131	0.128	0.124	0.125	0.126	0.119	0.12	0.115
$C_{1}$	0.127	0.000	0.000	0.000	0.000	0.143	0.165	0.153	0.146	0.153	0.137	0.131	0.132	0.133	0.126	0.127	0.122
$C_{2}$	0.000	0.000	0.000	0.000	0.000	0.000	0.138	0.138	0.132	0.138	0.135	0.000	0.000	0.000	0.000	0.000	0.000
$D_{1}$	0.119	0.000	0.000	0.000	0.000	0.121	0.000	0.155	0.148	0.155	0.152	0.122	0.123	0.124	0.000	0.000	0.112
$D_{2}$	0.121	0.000	0.000	0.000	0.000	0.122	0.147	0.000	0.151	0.159	0.156	0.140	0.142	0.143	0.122	0.123	0.117
$E_{}$	0.000	0.000	0.000	0.000	0.000	0.115	0.139	0.151	0.000	0.151	0.138	0.133	0.134	0.135	0.115	0.116	0.112
$F_{}$	0.000	0.000	0.000	0.000	0.000	0.000	0.142	0.154	0.146	0.000	0.151	0.136	0.138	0.139	0.132	0.133	0.115
$G_{}$	0.000	0.000	0.000	0.000	0.000	0.000	0.137	0.149	0.130	0.149	0.000	0.132	0.133	0.134	0.113	0.114	0.110
$H_{1}$	0.000	0.000	0.000	0.000	0.000	0.000	0.141	0.141	0.119	0.141	0.139	0.000	0.150	0.151	0.145	0.147	0.142
$H_{2}$	0.000	0.000	0.000	0.000	0.000	0.000	0.140	0.140	0.118	0.140	0.138	0.136	0.000	0.150	0.144	0.145	0.140
$H_{3}$	0.000	0.000	0.000	0.000	0.000	0.000	0.139	0.138	0.117	0.138	0.137	0.135	0.136	0.000	0.143	0.144	0.139
$I_{1}$	0.000	0.000	0.000	0.000	0.000	0.000	0.134	0.119	0.112	0.119	0.117	0.129	0.130	0.131	0.000	0.139	0.135
$I_{2}$	0.000	0.000	0.000	0.000	0.000	0.000	0.132	0.118	0.111	0.118	0.116	0.128	0.129	0.130	0.125	0.000	0.133
$J_{}$	0.000	0.000	0.000	0.000	0.000	0.000	0.114	0.113	0.000	0.113	0.111	0.125	0.126	0.127	0.134	0.135	0.000

**Table 6:** Results of the fuzzy DEMATEL application.

Equipment	*D*	*R*	D+R	D−R
$A_{}$	1.774	2.656	4.430	0.881
$B_{1}$	0.563	1.996	2.559	1.433
$B_{2}$	0.903	1.846	2.749	0.944
$B_{3}$	0.934	1.913	2.847	0.980
$C_{1}$	1.080	2.085	3.164	1.005
$C_{2}$	1.823	1.752	3.576	−0.071
$D_{1}$	2.366	1.842	4.208	−0.524
$D_{2}$	2.353	1.926	4.279	−0.427
$E_{}$	2.171	1.759	3.930	−0.412
$F_{}$	2.351	1.821	4.172	−0.530
$G_{}$	2.287	1.719	4.006	−0.568
$H_{1}$	2.190	1.826	4.016	−0.365
$H_{2}$	2.221	1.798	4.019	−0.423
$H_{3}$	2.252	1.771	4.024	−0.481
$I_{1}$	2.112	1.663	3.775	−0.449
$I_{2}$	2.142	1.637	3.780	−0.505
$J_{}$	2.057	1.568	3.625	−0.490

## Data Availability

Not applicable.

## Abbreviations

The following abbreviations are used in this manuscript:

AI	Artificial Intelligence
AR	Augmented Reality
BWM	Best Worst Method
CFSC	Converting Fuzzy Data into Crisp Scores
DEMATEL	DEcision-MAking Trial and Evaluation Laboratory
GA	Genetic Algorithm
IoT	Internet of Things
ISM	Interpretive Structural Modeling
MCDM	Multi-Criteria Decision-Making
ML	Machine Learning
NRM	Network Relationship Map
PSO	Particle Swarm Optimization
TFN	Triangular Fuzzy Number
